# Comparative Studies of Oleaginous Fungal Strains (*Mucor circinelloides* and *Trichoderma reesei*) for Effective Wastewater Treatment and Bio-Oil Production

**DOI:** 10.1155/2014/479370

**Published:** 2014-11-02

**Authors:** Anshuman Bhanja, Gauri Minde, Sandip Magdum, V. Kalyanraman

**Affiliations:** ^1^Department Biotechnology, Indian Institute of Technology Guwahati, Guwahati 781039, India; ^2^COE Biotechnology, R.D. Aga Research, Technology and Innovation Centre, Thermax Ltd., Pune 411019, India

## Abstract

Biological wastewater treatment typically requires the use of bacteria for degradation of carbonaceous and nitrogenous compounds present in wastewater. The high lipid containing biomass can be used to extract oil and the contents can be termed as bio-oil (or biodiesel or myco-diesel after transesterification). The separate experiments were conducted on actual wastewater samples with 5% v/v inoculum of* Mucor circinelloides* MTCC1297 and* Trichoderma reesei* NCIM992 strains. The observed reductions in chemical oxygen demand (COD) were 88.72% and 86.75% in 96 hrs and the observed substrate based biomass yields were 0.21 mg VSS/mg COD and 0.22 mg VSS/mg COD for* M. circinelloides *reactor and for* T. reesei* reactor, respectively. The resulted bio-oil production from wastewater treatment by* M. circinelloides* and* T. reesei* reactors was 142.2 mg/L and 74.1 mg/L, whereas biomass containing bio-oil contents (%w/w) were 22.11% and 9.82%, respectively. In this experiment, the fungal wastewater treatment was also compared with conventional bacterial process with respect to specific growth rate, biomass production, and oil content. This study suggests that wastewater can be used as a potential feedstock for bio-oil production with the use of oleaginous fungal strains and which could be a possible route of waste to energy.

## 1. Introduction

The efficient use of organic cycle via waste to energy configuration can contribute to reducing the current energy crisis. Numbers of countries in the world, including India, are currently passing through the critical phase of population explosion and the growing population demands more energy inputs [1]. Therefore, to ensure long-term sustainability, suitable alternative methods for oil production have to be developed which can be used as feedstock for several industrial applications [2]. Various wastes to energy conversion possibilities were explored till date, like kitchen waste to biogas [3], plant/agriculture waste to alcohol [4], anaerobic wastewater treatment to biogas [5], and wastewater treatment by using microbial fuel cell to produce hydrogen and electricity [6].

Basically, bacterial system is ideal for wastewater treatment due to its high rate of organic degradation. In case of yeast, they have fast growth rate compared to fungi and also found the oil assimilating capacity; in the recent study, researchers successfully used high strength brewery effluents for the production of microbial lipids for biodiesel production by using yeast* Rhodotorula glutinis* [7]. A similar study was effectively carried out by using high organic strength starch wastewater treated with the same yeast,* Rhodotorula glutinis*. Some other types of yeast also reported for high strength wastewater treatment with bio-oil production, such as* Trichosporon dermatis* and* Rhodosporidium toruloides* Y2, were used to treat wastewater from ethanol fermentation [8, 9]. But use of sewage wastewater or low strength wastewater for bio-oil or biodiesel production by using yeast or fungi has not been very well reported. Fungi are also known for their efficient carbon reduction and responsible for degradation of various tough to degrade substrates, such as polyvinyl alcohol [10]. There have been a few explorations into this technology, but most of them aim at growing fungi in nutrient media or synthetic substrates and then extracting lipids through them [11, 12]. Due to some characteristics like slow growth rate compared to bacteria (faster than algae), acidic pH requirement, filamentous growth, and spore forming nature, not many researchers have conducted experiments by using fungi to produce lipids from wastewater and hardly any commercial options are available. Some studies of high organic concentrations effluent were carried out for fungal bio-oil production.

Hence, not much research has gone into fungal wastewater treatment despite the lipid production being an advantage. Certain strains, such as* Aspergillus oryzae*,* Rhodosporidium toruloides*,* Epicoccum purpurascens*,* Lipomyces starkeyi*,* Trichosporon dermatis*,* Mortierella isabellina,* and* Mucor circinelloides*, are known to accumulate high amounts of lipids and the lipid content ranging between 18.1 and 42.6% of their biomass and are thus called oleaginous strains [13–20]. The fatty acid profiles of microbial bio-oils are quite similar to those of conventional vegetable oils; hence, oleaginous filamentous fungi are suggested as a favorable feedstock for a sustainable biodiesel industry [15, 21]. In this context, the wastewater can be considered as a cost-effective candidate of raw materials for biodiesel production [22]. Biodiesel has the ability to mix with petroleum diesel in any ratio and still function; biodiesel can be considered as a clean and renewable first alternative solution to oily energy resource [23].

This work aims at producing value added product in the form of bio-oil which would be an energy source produced in parallel with fungi based wastewater treatment. The fungal wastewater treatment can overcome the sludge handling as one of the disadvantages of the traditional bacterial wastewater treatment.

## 2. Materials and Methods

### 2.1. Wastewater Sample and Inoculum Preparation

10 liters of wastewater sample was collected from the equalization tank of ETP present in factory premises of Thermax Ltd. located at Pune, India. The collected wastewater sample was analyzed for COD, TOC, pH, NH3-N, and phosphate.* Mucor circinelloides* (MTCC1297; Microbial Type Culture Collection, India) and* Trichoderma reesei* (NCIM992; National Collection of Industrial Microorganisms, India) were selected and collected as inoculum as they are reported to be high lipid yielding fungi. Two sets of 500 mL Sabouraud Agar broth (HiMedia Ltd.) (30 gm of dry Sabouraud Agar broth in 1 L distilled water, pH 5.8) in 1 lit conical flasks separately were autoclaved and cooled in water bath to room temperature. Further, the flasks containing broths were inoculated separately with lyophilized cultures of* M. circinelloides* and* T. reesei*. The two flasks were left on a rotary shaker at 120 RPM speed for 6 days at room temperature for both fungal cultures to grow as inoculum. The bacterial sludge was collected from tube settler out of nearby MBBR plant as inoculum for third reactor for comparison with conventional bacterial wastewater process.

### 2.2. Experimentation

The pH was reduced to 4.97 of actual wastewater (6 liter) with COD of 2862.16 mg/L being taken. This was divided into three equal portions and poured into three bottles with 3 liter volume. Aeration pipes with bubbler stones were inserted and the bottles were cotton plugged with cotton and the three systems were autoclaved for 15 minutes at 110°C with 15 psi pressure to avoid bacterial contamination and then cooled to room temperature. Since the fungal inoculums were incubated on a rotary shaker, the fungal flocks formed, which were roughly spherical masses of different sizes ranging from 0.6 to 0.9 cm. The 100 mL of inoculums of the two fungal strains (*M. circinelloides* and* T. reesei*) and bacterial sludge was centrifuged and washed repeatedly with autoclaved distilled water and the same was inoculated into their respective 3 liter bottles containing wastewater as a substrate aseptically. The reactors were started with aeration rate 8 LPM and reactor samples were obtained on 1st, 2nd, 3rd, and 4th days. The samples were filtered and the TOC was determined and the pH was recorded with a Jenco 6230M pH meter (Jenco Instruments, San Diego, CA) for each sample. The samples from fungal reactors were analyzed under a microscope with 40x magnification (Dewinter Trinocular Microscope-Model: Select) to check for bacteria contaminations.

### 2.3. Bio-Oil Extraction

The fungal and bacterial biomasses from each bioreactor were collected and dried at 60°C. The samples of dry masses were then crushed with mortar and pestle and added to 10 mL of distilled water. 2 mL 1 : 1 sulphuric acid was added and then 25 mL n-hexane was added to the mixture to dissolve the lipids. The solutions were left on a rotary shaker overnight. The layer of hexane was extracted out and filtered through a 40 Whatman filter paper with 6 gm of sodium sulphate. The filtrate was then poured into a dry crucible (whose dry weight was measured) and heated at 60°C for the hexane to vaporize leaving only the lipid. The total weight was measured. The oil extraction procedure was repeated for all the fungal and bacterial biomasses. The weight differences for the respective fungal and bacterial samples were recorded.

### 2.4. Analytical Methods

The COD (APHA method 5220B), NH3-N (APHA method number: 4500-NH3 E), and phosphate (APHA method: 4500-P C) of water samples were used according to the procedure described in Standard Methods [24]. Total suspended solids and fixed and volatile solids were analyzed by procedure described in Standard Methods (APHA method: 2540 D & E). The total organic carbon (TOC) analysis of all samples was carried by TOC-V CPH (Shimadzu Ltd.) with multiplying factor of 2.66 for final indirect COD quantification [25].

## 3. Results and Discussions

### 3.1. Fungal Organic and Nutrient Reduction

**Figure 1:**
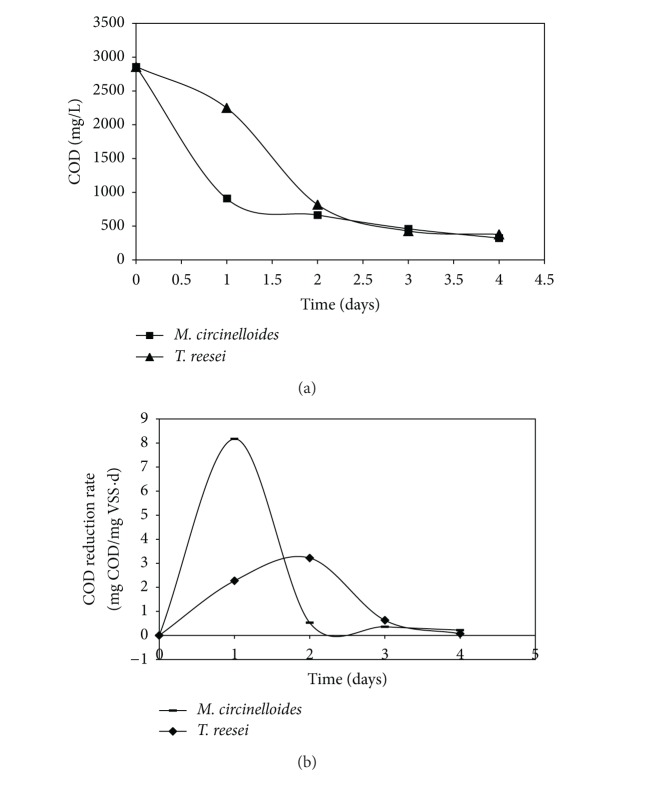
COD reduction and rate profile during fungal wastewater treatement. (a) Effect of different fungal strain inoculums (*M. circinelloides *and* T. reesei*) on COD reduction profile and (b) effect of different fungal strain inoculum on COD reduction rates.

Initial COD, NH3-N, and phosphate concentrations of wastewater sample with inoculum were 2862.16 mg/L, 41.5 mg/L, and 17.38 mg/L, respectively. The percentage COD reductions were 68.38% and 21.5% for* M. circinelloides* inoculated and* T. reesei* inoculated reactors on first day, respectively (Figure 1(a)). Whereas The COD reduction rates on first and second day of TR were 2.27 mg COD/mg VSS*·*d and 3.22 mg COD/mg VSS*·*d, respectively, indicating slightly increased respiratory activity on second day (Figure 1(b)). It indicates that* M. circinelloides* reduces COD, probably rbCOD at higher rate than* T. reesei*, which reduces COD at slower but consistent rates. By the end of the fourth day of batch process, the COD, NH3-N, and phosphates of* M. circinelloides* and* T. reesei* reactor samples were 322.92 mg/L, 4.66 mg/L, and 2.08 mg/L and 379.32 mg/L, 5.89 mg/L, and 3.67 mg/L, respectively.

### 3.2. Fungal Biomass Production

**Figure 2:**
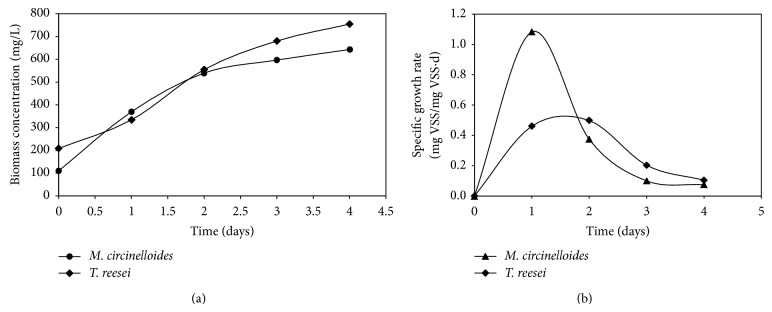
Fungal biomass production and specific growth rates profile during wastewater treatement. (a) Effect of different fungal strain inoculum (*M. circinelloides* and* T. reesei*) on biomass generation profile and (b) effect of different fungal strain inoculum on specific growth rates.

**Figure 3:**
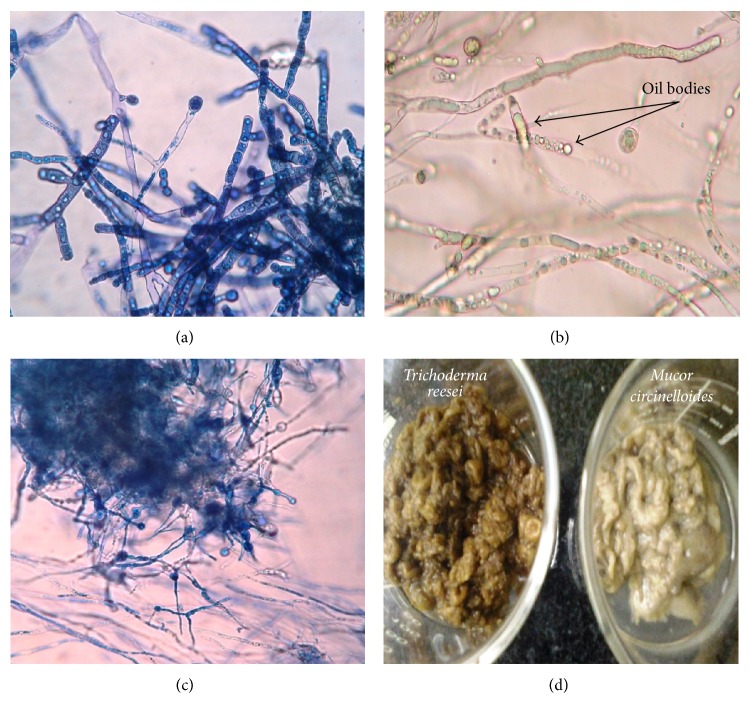
Microscopic observation of (a)* M. circinelloides* filamentous structure (Cotton Blue staining), (b) oil/lipid bodies stored in mycelia of* M. circinelloides,* (c)* T. reesei* filamentous structure (Cotton Blue staining), (d) fungal biomass collected at the end of the wastewater treatment (Left:* T. reesei* and right:* M. circinelloides*).

After inoculation of* M. circinelloides *and* T. reesei *to their respective reactors containing wastewater, their initial VSS was 109.86 mg/L and 208.57 mg/L, respectively (Figure 2(a)). The biomass yield of* T. reesei *was 0.22 mg VSS/mg COD, which was more than* M. circinelloides *yield (0.21 mg VSS/mg COD), probably due to higher initial biomass concentration. The final VSS concentrations of* M. circinelloides *and* T. reesei *reactors were 643.1 mg/L and 754.8 mg/L, which were less than VSS yield of 1.36 gm/L at high glucose concentration reported by Xia et al. [20]. Another study of* M. isabellina* with glucose being a substrate resulted in 17.5 g/L of biomass, containing 12.7 g/L of lipid, with lipids representing about 90–92% w/w [31]. This represents the use of simple sugars favored by fungi for its growth. The specific growth rates of* M. circinelloides* and* T. reesei* reactors were compared and found to be 1.083 mg VSS/mg VSS*·*d and 0.461 mg VSS/mg VSS*·*d, respectively, after 24 hrs (Figure 2(b)). The specific growth rate for* M. circinelloides *reactor gradually decreased and it was 0.075 mg VSS/mg VSS*·*d at the end of the fourth day, whereas, in case of the* T. reesei *reactor, the specific growth rate was increased to 0.498 mg VSS/mg VSS*·*d on the 2nd day and further it decreased to 0.104 mg VSS/mg VSS*·*d at the fourth day. In the present experiment, although the* M. circinelloides *showed slightly lower biomass production rate, it had high specific growth rate compared to TR. The microscopy and biomass images of* M. circinelloides* and* T. reesei* were observed at day 4 (Figure 3).

### 3.3. Fungal Bio-Oil Production

At the end of wastewater treatment, the biomasses of* M. circinelloides* and* T. reesei* were used for bio-oil extraction. The net dry biomass weights collected from the* M. circinelloides* and* T. reesei* reactors were 1286.2 mg and 1509.6 mg, respectively, per two liter cultures. 284.43 mg and 148.2 mg were the oil recovered from extraction process from* M. circinelloides* and* T. reesei* dry biomasses. The present wastewater treatment produced bio-oil yields were 142.2 mg/L and 74.1 mg/L for* M. circinelloides* and* T. reesei *reactors, respectively. The bio-oil content (% w/w) was calculated, which was 22.11% and 9.82% for both* M. circinelloides* and* T. reesei *strains, respectively.* M. circinelloides* is reported with the lipid accumulation capacity of 25% of the cell biomass [32], whereas the fungal cultivation on thin stillage for 2 days in a 6 L airlift bioreactor, resulted in 92% increase in oil yield and 20 gm/L of dry fungal biomass with a lipid content of 46% (gm of oil per 100 g dry biomass) [33]. The study of liquid media containing high strength oil mill wastewater as sole carbon source showed that the* T. elegans* and* Z. moelleri* produced 4.4 and 3.5 gm/L cell mass in surface (SC) and submerged (SMC) cultures, respectively, containing around 60% (w/w) of lipids [34]. In the present study of low strength fungal wastewater treatment, the* M. circinelloides* was observed to be the highest oil yielding fungal strain in comparison with* T. reesei*. Different types of substrate used for bio-oil accumulation in respective fungus were compared in Table 1 for their oil generation ability.

### 3.4. Comparative Evaluation of the Fungal Processes with Conventional Bacterial Wastewater Degradation Study

Conventional bacterial process will always be a high rate degradation process. The comparative figures are shown in Table 2. The conventional bacterial wastewater treatment showed high maximum specific growth rate compared to both* M. circinelloides *and* T. reesei*. The conventional bacterial process has taken 48 hours to cross the 90% COD reduction from 2862.16 mg/L to 280.90 mg/L where as both fungal had not reached at the end of fourth day. So the conventional bacteria process shows maximum COD reduction in minimum time. Similarly, the total biomass production was higher in the conventional bacterial process than with the fungal process. But fungi could offer the benefit over bacteria in wastewater treatment processes, as the biomass produced during fungal wastewater treatment has, potentially, a much higher value than that from the conventional bacterial activated sludge process [35]. The bio-oil content in conventional bacterial process was nearly seven times lesser than* M. circinelloides *and three times less than* T. reesei.*

### 3.5. Cost Benefits Analysis

A different form of biomass with a higher value could significantly change the economics of wastewater treatment [35]. Only saponifiable lipids and free fatty acids could be produced to fatty acid methyl esters (FAMEs) which are suitable for biodiesel production. It is reported that 98% of the total lipids extracted from* M. circinelloides* were saponifiable lipids and free fatty acids and the oil composition met the specifications of the current existing standards of hydrocarbons [36]. While estimating the potential of wastewater to bio-oil synthesis for biodiesel production via fungal (*M. circinelloides*) route, the 100 m^3^/day capacity plant having wastewater with similar characteristics can produce 14.22 kg of bio-oil per day and 200 MLD plant can produce 28.44 tons of bio-oil per day (Table 3). Considering the above mentioned 98% saponifiable lipids content with 0.87 ton/m^3^ density of biodiesel, the theoretical biodiesel production will be 4.23 gal/day and 8436.87 gal/day with potential worth of 12.57 $/day and 25137.7 $/day for 100 m^3^/day and 200 MLD plant, respectively (calculation is based on reported B100 price of 2.97 $/gal [37]).

It shows the great opportunity for future sustainable wastewater treatment and bioenergy production at higher scale with high capacity plants. The further study needs to be done in the area of heterotrophic oleaginous fungal species and their fatty acids, such as triacylglycerols and sterol biosynthesis profiles with biochemical and genetic approaches for better understanding of their roles and regulations for future waste to energy applications.

## 4. Conclusion

Most aerobic wastewater treatment systems are energy intensive; the sludge produced from these systems creates the disposal problems, which need extra attention and expenses. The proposed fungal wastewater treatment technology offers very good waste to energy route by using low strength wastewater as potential substrate. The studied comparison of* M. circinelloides* and* T. reesei* for efficient wastewater treatment and bio-oil production found that the* M. circinelloides* was the best fungus with 88.72% of COD degradation ability with higher rate of degradation of 8.17 mg COD/mg VSS*·*d and bio-oil content 22.11% w/w. Further optimization strategies of using modified or different fungal strains may increase the efficiency of the proposed route. The wastewater treatment with energy generation technology could be a sustainable model for future to achieve high treatment efficiency and bioresource.

## Figures and Tables

**Table 1:** Different substrates used and bio-oil content in respective fungus were compared.

Substrate type	Fungi cultivated	Lipid accumulation (wt/wt)	Ref.
Glucose, with N limitation and xylose induction	*Cunninghamella echinulata *ATHUM 4411	57.70%	[26]
	*Mortierella isabellina *ATHUM 2935	65.50%	
Lactose	*Mortierella isabellina *	36%	[27]
Glucose, fructose, and sucrose	*Thamnidium elegans *CCF-1465	70%	[28]
Sweet sorghum extract	*Mortierella isabellina *	51%	[29]
Rice hull hydrolysate	*Mortierella isabellina *	64.30%	[30]
Low strength wastewater	*Mucor circinelloides MTCC1297 *	22.11%	Present study
	*Trichoderma reesei NCIM992 *	9.82%	

**Table 2:** Comparison of maximum specific growth rate, % COD reduction, biomass produced, and bio-oil content for different process inoculums.

Wastewater process inoculum type	Maximum specific growth rate	Time required for 90% COD reduction	Total biomass produced	Bio-oil content
	mg VSS/mg VSS*·*d	hrs	gm/lit	% w/w
*Mucor circinelloides *	1.083	>96	0.60	22.11
*Trichoderma reesei *	0.498	>96	0.68	9.82
Conventional bacterial process^*^	1.343	48	1.55	3.21

^*^The bacterial inoculum used in this process was collected from tube settler of MBBR plant, containing mixed bacterial flora.

**Table 3:** Cost benefit analysis of fungal route for wastewater treatment.

Plant capacity	*Mucor circinelloides *	*Trichoderma reesei *	Biodiesel production	Profits in $
MLD	Kg/day	Kg/day	Gal	Biodiesel rate (2.97 $/gal)
0.1	14.2215	7.41	4.23	12.57
1	142.215	74.1	42.32	125.69
10	1422.15	741	423.19	1256.88
200	28443	14820	8463.87	25137.7
